# Distinct roles for the hypoxia-inducible transcription factors HIF-1α and HIF-2α in human osteoclast formation and function

**DOI:** 10.1038/s41598-020-78003-z

**Published:** 2020-12-03

**Authors:** Helen J. Knowles

**Affiliations:** Botnar Research Centre, Nuffield Department of Orthopaedics Rheumatology & Musculoskeletal Sciences, University of Oxford, Oxford, UK

**Keywords:** Bone, Cell biology

## Abstract

Bone homeostasis is maintained by a balance between osteoblast-mediated bone formation and osteoclast-driven bone resorption. Hypoxia modulates this relationship partially via direct and indirect effects of the hypoxia-inducible factor-1 alpha (HIF-1α) transcription factor on osteoclast formation and bone resorption. Little data is available on the role(s) of the HIF-2α isoform of HIF in osteoclast biology. Here we describe induction of HIF-1α and HIF-2α during the differentiation of human CD14+ monocytes into osteoclasts. Knockdown of *HIF-1α* did not affect osteoclast differentiation but prevented the increase in bone resorption that occurs under hypoxic conditions. *HIF-2α* knockdown did not affect bone resorption but moderately inhibited osteoclast formation. Growth of osteoclasts in 3D gels reversed the effect of *HIF-2α* knockdown; *HIF-2α* siRNA increasing osteoclast formation in 3D. Glycolysis is the main HIF-regulated pathway that drives bone resorption. *HIF* knockdown only affected glucose uptake and bone resorption in hypoxic conditions. Inhibition of glycolysis with 2-deoxy-d-glucose (2-DG) reduced osteoclast formation and activity under both basal and hypoxic conditions, emphasising the importance of glycolytic metabolism in osteoclast biology. In summary, HIF-1α and HIF-2α play different but overlapping roles in osteoclast biology, highlighting the importance of the HIF pathway as a potential therapeutic target in osteolytic disease.

## Introduction

Bone homeostasis is maintained throughout life by a balance between osteoblast-mediated bone formation and osteoclast-driven bone resorption. Hypoxia modulates this homeostatic relationship during development, in response to mechanical trauma (e.g. fracture) and in bone pathologies such as osteoporosis, rheumatoid arthritis and cancer, via the hypoxia-inducible factor (HIF) transcription factor.

HIF is a heterodimer regulated by the stability of its inducible alpha subunits (HIF-1α, HIF-2α). Under standard conditions, HIFα is post‐translationally hydroxylated at two conserved proline residues by the prolyl‐4‐hydroxylase enzymes (PHD1–3), leading to interaction with the von Hippel-Lindau (VHL) protein, polyubiquitination and proteasomal degradation. Separately, hydroxylation of an asparagine residue by Factor Inhibiting HIF-1 (FIH) prevents its transcriptional activity^1^. Hypoxia reduces the activity of the O_2_-dependent PHD enzymes, allowing nuclear accumulation of HIFα and enabling its association with HIFβ and other co-factors. HIF transcriptional activity is simultaneously increased due to reduced asparaginyl hydroxylation by FIH, allowing it to bind the hypoxia‐response element (HRE) in the promoters of HIF target genes to initiate transcription^1–3^.

Most studies describing the roles of the PHD enzymes and HIF in bone focus on HIF-1α and there is very little direct data on the role(s) of HIF-2α. HIF-2α regulates different but overlapping genes to HIF-1α and modulates the HIF transcriptional response in a microenvironment- and tissue-specific manner^4,5^. Mice with an osteoblast-specific deletion of *Vhl*, resulting in overexpression of both HIF isoforms, produce abundant vascular endothelial growth factor (VEGF) and develop extremely dense, highly vascularized long bones^6^. Osteoblast-specific *Hif-1α* knockout mice demonstrate the reverse skeletal phenotype: decreased trabecular bone volume, reduced osteoblast number and bone formation rate and decreased vascularity. In contrast, mice lacking *Hif-2α* have only a modest decrease in trabecular bone volume, despite exhibiting an equivalent reduction in blood vessel development within the long bones. This suggests that HIF-1α and HIF-2α exert both distinct and overlapping functions in long bone development; HIF-1α alone exerting direct effects on osteoblasts while both isoforms promote vascularisation of bone^6,7^. Recently, however, heterozygous *Hif-2α*^+*/–*^ knockout mice and *Hif-2αfl/fl;Col1a1-Cre* mice with an osteoblast-specific depletion of *Hif-2α* were shown to exhibit increased bone mass associated with increased numbers of osteoblasts, due to loss of a HIF-2α -mediated inhibition of osteoblast differentiation that increases TWIST2 expression to downregulate osteoblast-associated osteocalcin and RUNX2^8^. The reason for the difference between these observations is unclear.

As well as driving osteogenic-angiogenic coupling^9^, HIF also affects bone-resorbing osteoclasts. Osteoclasts form by the fusion of CD14+ monocytic precursors, induced by the cytokines macrophage colony-stimulating factor (M-CSF) and receptor activator of NFκB ligand (RANKL), to produce multinucleated bone-resorbing cells^10,11^. Much data describes indirect effects of HIF to stimulate osteoclast formation and activity via regulation of the expression of osteoclastogenic factors by osteoblasts and surrounding stromal cells^12^. For example, HIF-induced lysyl oxidase produced by tumour cells stimulates osteoclast differentiation, although whether this is via stimulation of RANKL production by osteoblasts or a direct RANKL-independent mechanism remains unclear^13,14^. Similarly, HIF-induced angiopoietin-like 4 produced by stromal cells directly stimulates bone resorption by osteoclasts^15^. Hypoxia and HIF also exert direct effects on osteoclasts. By directly comparing hypoxia, *HIF* knockdown, HIF induction and *PHD* enzyme depletion, we and others have shown that PHD2 drives bone resorption by mature osteoclasts via induction of HIF-1α^12,16–18^. Osteoclast-specific inactivation of *HIF-1α* antagonises osteoporotic bone loss in mice, suggesting that HIF-1α also directly promotes osteoclast activation and bone loss in vivo^19^.

The majority of these reports focus on either the HIF pathway in general or HIF-1α; there is very little data specifically on the role of HIF-2α in osteoclast biology, most of which refers to indirect effects of HIF-2α via its effects on osteoblasts and surrounding stromal cells. Wu et al. showed that mice with an osteoblast-specific mutation in *Phd2/3* have fewer osteoclasts in vivo and a high bone mass due to HIF-2α-mediated induction of osteoprotegerin (OPG), an inhibitor of osteoclast formation and activity^20^. Elevated serum concentrations of OPG also occur in *Phd3*^*−/−*^ mice associated with reduced serum CTXI, indicative of reduced osteoclast activity^17^. However, Bae et al. described the opposite effect in periodontal ligament cells (PDLCs). HIF is upregulated in the chronically inflamed PDLCs of periodontitis patients and by nicotine- and LPS-exposed PDLC in vitro. The conditioned medium produced by nicotine and LPS-treated PDLCs increases the number of osteoclasts that form by differentiation of murine bone marrow-derived macrophages (BMMs). This increase is blocked by siRNA targeting *HIF-2α*, but not *HIF-1α*, via reduced production of inflammatory cytokines by the PDLCs^21^. Similarly, Rauner et al.showed that conditional deletion of *Phd2* in CD68-expressing monocytes/macrophages causes reduced bone mass. This phenotype was rescued by additional conditional deletion of *Hif-2α*, but not *Hif-1α*, leading to the discovery that *Phd2* depletion stabilises HIF‐2α and causes chronic induction of erythropoietin which inhibits the differentiation and mineralization of osteoblast progenitors and stimulates osteoclast formation, resulting in lower bone density^22^. Lee et al. showed that *Hif-2α* deficiency in mice enhances bone mass, partially because HIF-2α directly induces RANKL expression in osteoblasts leading to increased osteoclastogenesis^8^. HIF-2α-mediated induction of RANKL also occurs in fibroblast-like synoviocytes from the inflamed synovium in rheumatoid arthritis^23^.

Overall, the limited data on paracrine effects of HIF-2α suggests that it generally stimulates osteoclast formation and activity. This is supported by the only study to date looking at direct effects of HIF-2α in osteoclasts. Lee et al.showed that over-expression of *Hif-2α* in murine BMMs enhanced osteoclast differentiation and the expression of osteoclast-specific genes, whereas reduced osteoclastogenesis was evident in BMMs from *Hif-2α*^+*/−*^ mice and those treated with a specific inhibitor of HIF-2α. This is due to direct regulation of Traf6, a central component of the osteoclastogenic RANK signalling pathway, by HIF-2α^8^.

Given the potential of manipulation of the HIF pathway as a therapeutic strategy to improve bone formation and/or reduce bone loss, it is essential that we improve our understanding of the effects of specific isoforms of HIF on osteoclast formation and function. This is especially important as current HIF inhibitors are largely not specific for the HIF pathway^24^, meaning that therapeutic siRNA may represent a more targeted strategy^25,26^. Here, we present the first preliminary investigation of the direct and specific effects of HIF-2α on the differentiation and bone resorption capacity of human osteoclasts.

## Results

### HIF-1α and HIF-2α are induced during osteoclast differentiation

**Figure 1:**
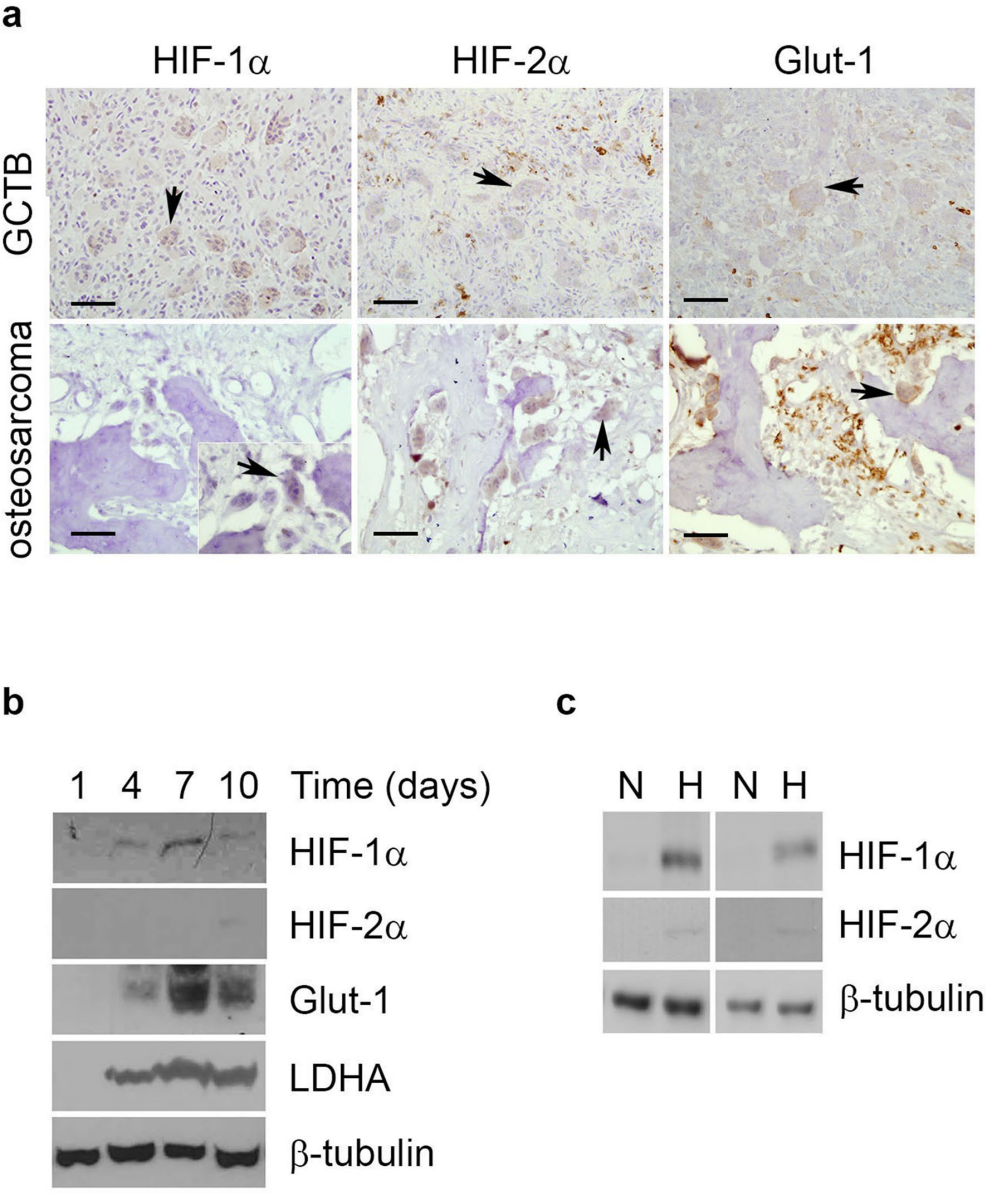
HIF-1α and HIF-2α are induced during osteoclast differentiation. (**a**) Immunohistochemistry for HIF-1α, HIF-2α and Glut-1 in representative sections from GCTB and osteosarcoma tumours containing visible osteoclasts. Scale bar = 50 µm. Arrows indicate multinucleated osteoclasts. (**b**) Western blot showing stabilisation of HIF-1α and HIF-2α protein and induction of Glut-1 and LDHA in comparison to the β-tubulin loading control during the timecourse of differentiation of CD14+ human monocytes into osteoclasts. (**c**) Western blot showing stabilisation of HIF-1α and HIF-2α protein in comparison to the β-tubulin loading control in mature human osteoclasts exposed to hypoxia (H, 2% O_2_) for 24 h versus the corresponding normoxic (N) control. Full-length blots are presented in Supplementary Figure 1.

Osteoclasts are often present in pathological conditions that affect bone and are particularly numerous in primary bone sarcomas. Both HIF-1α and HIF-2α proteins are expressed in osteosarcoma and giant cell tumour of bone (GCTB), as well as HIF-regulated Glut-1^27–29^. Expression of both HIF isoforms can be observed in multi-nucleated osteoclasts when the surrounding tumour cells exhibit visibly lower expression of both HIF and Glut-1 (Fig. 1a), suggesting that expression of HIF in osteoclasts may be independent of microenvironmental hypoxia. We and others have previously described induction of HIF-1α during osteoclastogenesis induced with M-CSF and RANKL^17,19,27,30^. HIF-1α, HIF-2α and the HIF-regulated proteins Glut-1 and lactate dehydrogenase A (LDHA) were induced during the differentiation of CD14+ human monocytes into osteoclasts (Fig. 1b). The amount of HIF-2α was only just within detectable limits, despite over-loading of the Western blot. This may be due to reduced basal expression of this isoform of HIF in osteoclasts, as was observed following hypoxic exposure (Fig. 1c).

### Digoxin inhibits osteoclast formation and activity

**Figure 2:**
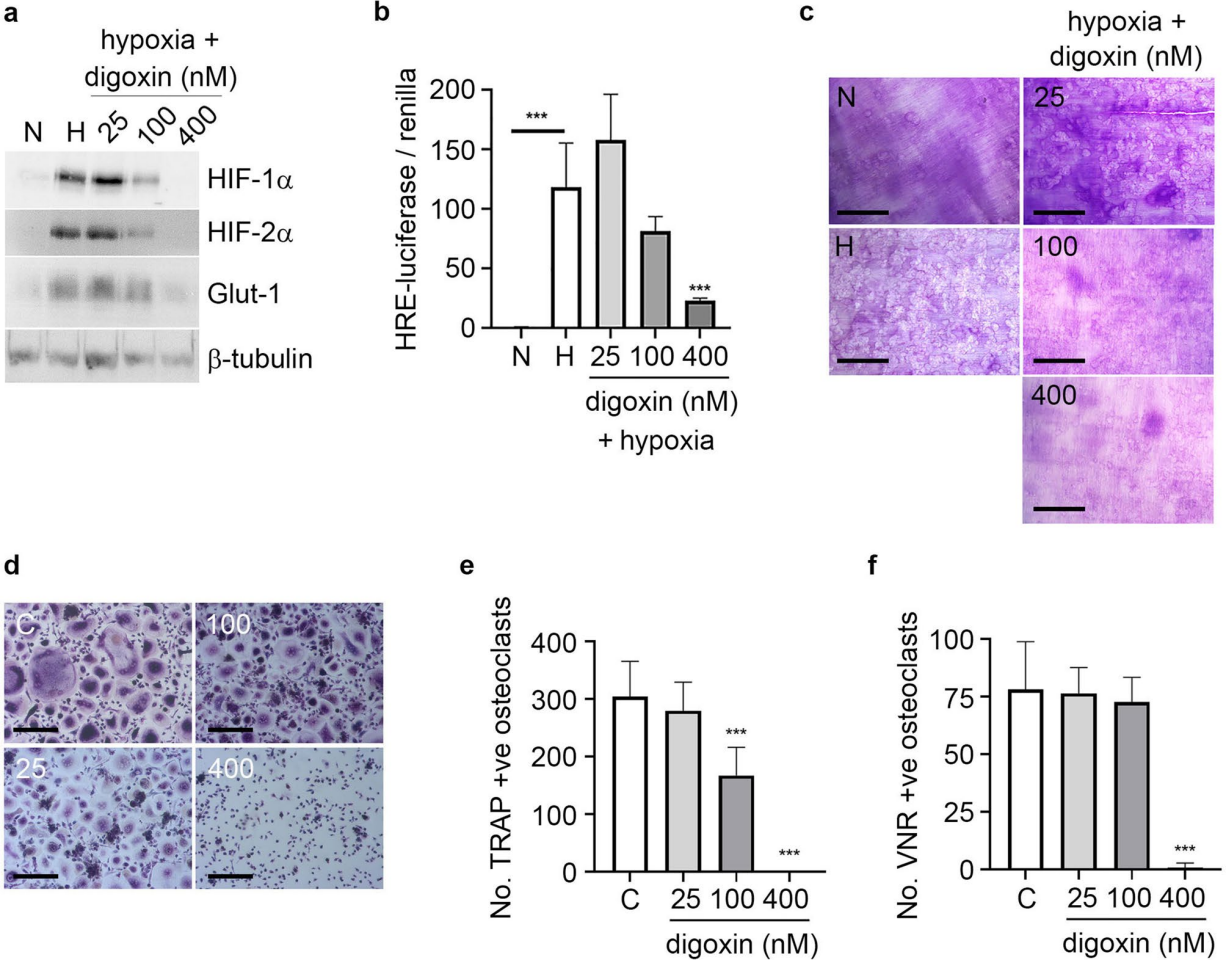
Digoxin inhibits osteoclast formation and activity. (**a**) Western blot showing expression of HIF-1α, HIF-2α and Glut-1 protein in comparison to the β-tubulin loading control; (**b**) HRE-luciferase activity (n = 4) and (**c**) representative images of toluidine blue-stained dentine discs revealing resorption tracks produced following 24 h exposure to hypoxia (H, 2% O_2_) plus 25–400 nM digoxin versus the corresponding normoxic (N) control. Scale bar = 700 µm. (**d**) Representative images and (**e**) quantification of TRAP-stained osteoclasts formed after 9 days of differentiation in the presence of 25–400 nM digoxin. Scale bar = 200 µm. n = 5. (**f**) Quantification of VNR-positive osteoclasts formed on dentine discs after differentiation under the same conditions. n = 5. ***p  < 0.001. Full-length blots are presented in Supplementary Figure 1.

Digoxin is a cardiac glycoside that inhibits the activity of sodium potassium adenosine triphosphatase (Na+/K+‐ATPase). It also affects translation of HIF-1α and HIF-2α, causing inhibition of the HIF pathway^31^. Digoxin was recently shown to inhibit osteoclastogenesis in murine monocytic RAW264.7 cells via inhibition of RANKL-induced HIF-1α during differentiation^32^. In human CD14+ monocyte-derived osteoclasts, digoxin showed equivalent and complete inhibition of hypoxia-induced HIF-1α and HIF-2α, as well as inhibition of HIF-induced Glut-1 and reduced hypoxic activation of the phosphoglycerate kinase (PGK)-HRE luciferase reporter construct (Fig. 2a,b). Digoxin exhibited dose-dependent inhibition of the hypoxia-induced increase in bone resorption activity, that was maximal at the 400 nM concentration at which both HIF isoforms were fully inhibited (Fig. 2c). Similarly, digoxin showed dose-dependent inhibition of osteoclast differentiation under basal conditions; completely inhibiting osteoclast formation at the highest concentration (Fig. 2d–f). However, as digoxin inhibits expression of both HIF-1α and HIF-2α these effects cannot be specifically attributed to inhibition of either isoform.

### HIF-2α has a moderate effect on osteoclast formation

**Figure 3:**
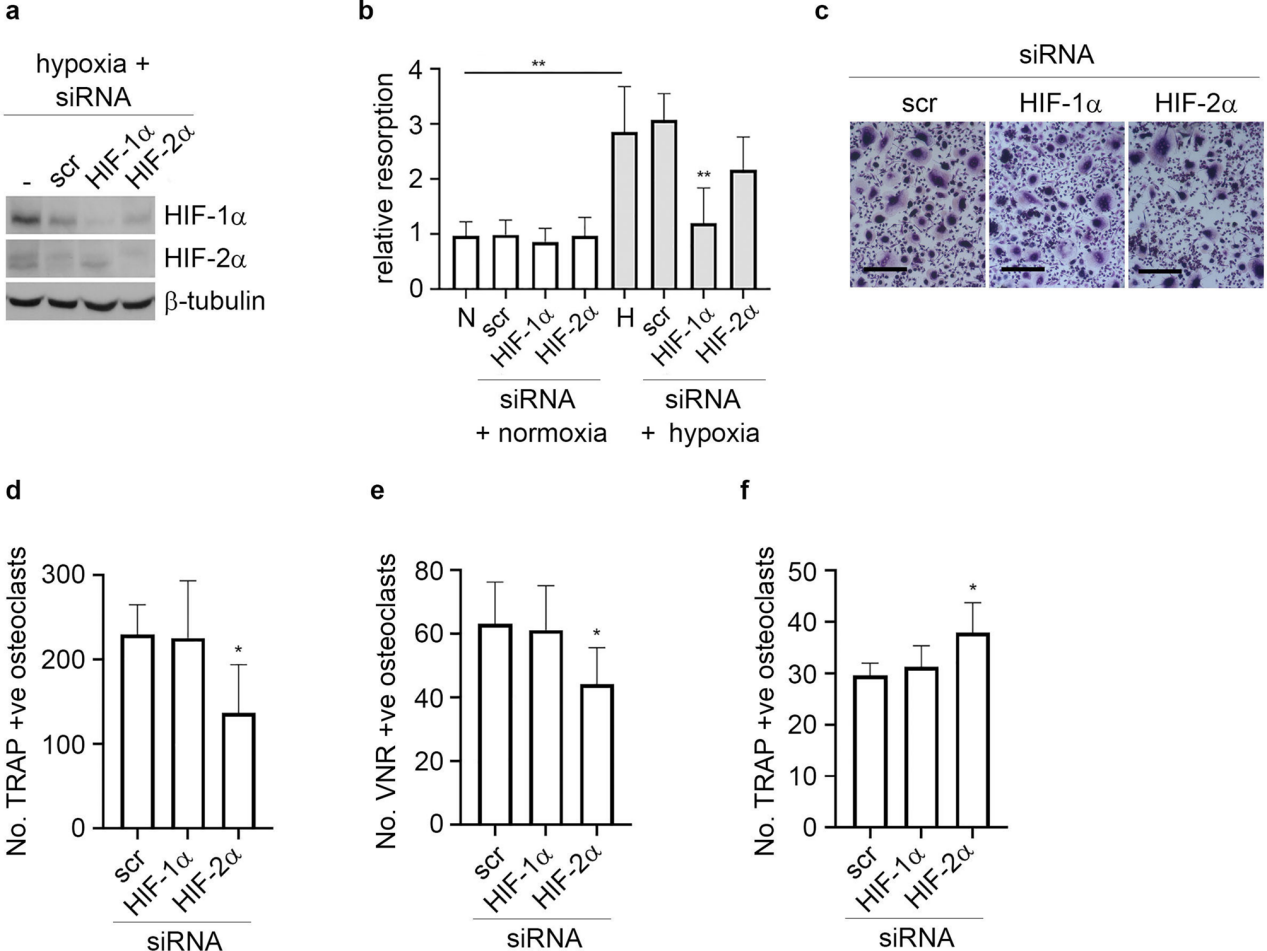
HIF-2α has a moderate effect on osteoclast formation. (**a**) Western blot showing expression of HIF-1α and HIF-2α protein in hypoxic osteoclasts (24 h, 2% O_2_) in comparison to the β-tubulin loading control following treatment of mature human osteoclasts with siRNA targeting *HIF-1α*, *HIF-2α* or a scrambled (scr) siRNA control. (**b**) Effect of the same treatments on the amount of bone resorption performed by mature human osteoclasts. n = 7. (**c**) Representative images and (**d**) quantification of TRAP-stained osteoclasts formed after 9 days of differentiation in the presence of siRNA targeting *HIF-1α*, *HIF-2α* or a scrambled (scr) siRNA control. Scale bar = 200 µm. n = 5. (**e**) Quantification of VNR-positive osteoclasts formed on dentine discs after differentiation under the same conditions. n = 6. (**f**) Number of TRAP-positive osteoclasts present 24 h after reseeding of osteoclasts generated in a 3D collagen gel in the presence of siRNA targeting *HIF-1α*, *HIF-2α* or a scrambled (scr) siRNA control. n = 4. *p < 0.05; **p < 0.01. Full-length blots are presented in Supplementary Figure 1.

We next used isoform-specific *HIF* siRNA to determine the effect of specific inhibition of either *HIF-1α* or *HIF-2α* on osteoclast formation and activity. Efficacy of knockdown was confirmed by Western blot in hypoxic osteoclasts (Fig. 3a). As we have previously reported^17^, knockdown of HIF-1α completely ablated the hypoxic increase in bone resorption (Fig. 3b) but had no effect on osteoclast differentiation (Fig. 3c–e). Conversely, knockdown of HIF-2α had a small inhibitory effect on osteoclast differentiation (Fig. 3c–e) but did not affect bone resorption (Fig. 3b). We recently reported that osteoclastogenesis can be differentially affected in 3D versus 2D culture^33^. In contrast to standard culture conditions, *HIF-2α* siRNA caused a moderate increase in osteoclast formation when cells were cultured in 3D collagen gels (Fig. 3f).

### Role of glycolysis in the differential effects of HIF-1α and HIF-2α

**Figure 4:**
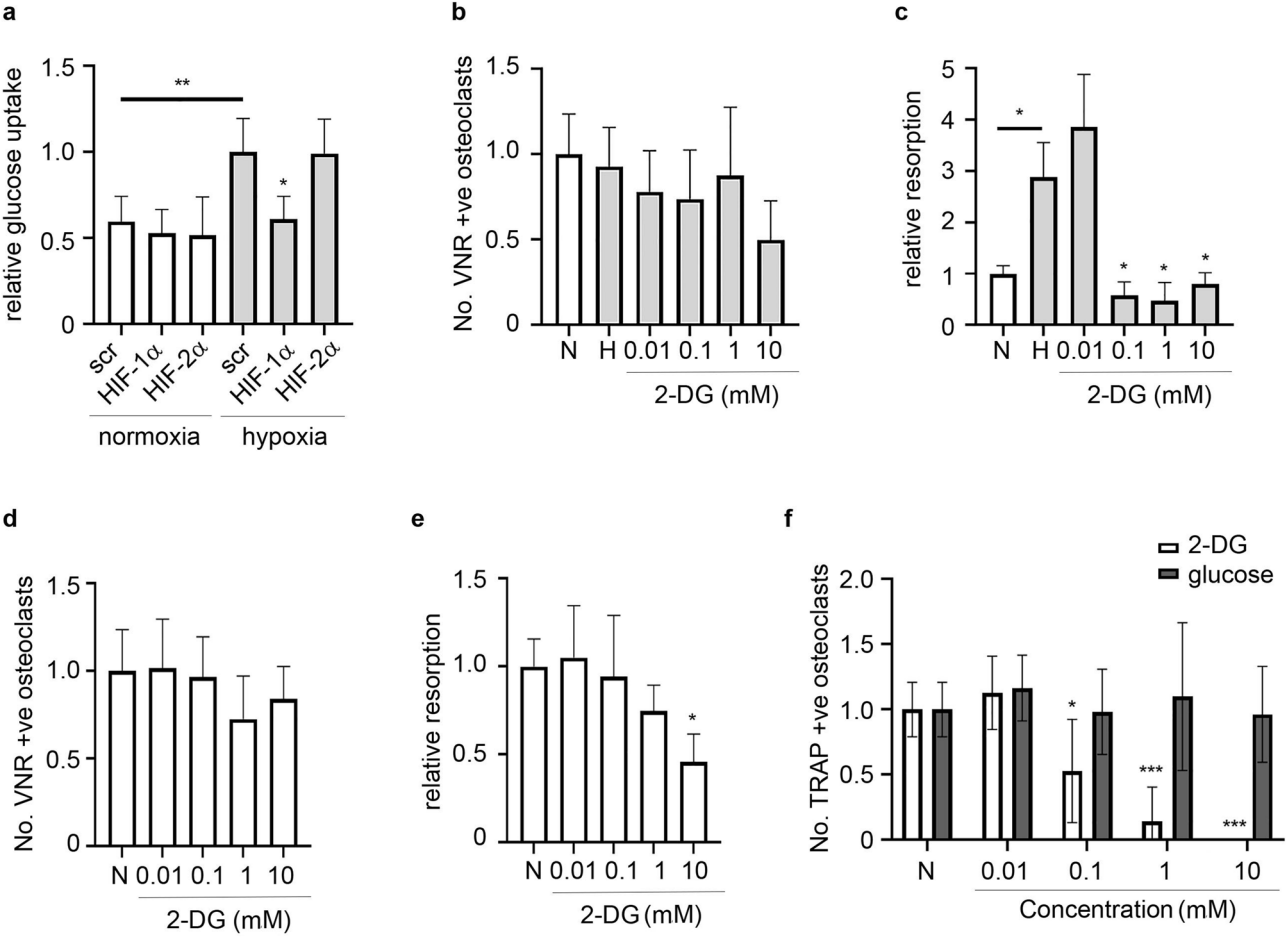
Role of glycolysis in the differential effects of HIF-1α and HIF-2α. (**a**) Relative glucose consumption following treatment of mature human osteoclasts with siRNA targeting *HIF-1α*, *HIF-2α* or a scrambled (scr) siRNA control and exposed to normoxia or hypoxia (2% O_2_) for 24 h. n = 6. (**b**,**d**) Number of VNR-positive osteoclasts and (**c**,**e**) amount of bone resorption performed by mature osteoclasts cultured on dentine discs and exposed to 2-DG (0.01–10 mM) under either (**b**,**c**) hypoxic (H, 2% O_2_) or (**d**,**e**) normoxic conditions for 24 h. n = 3–4. (**f**) Number of TRAP-positive osteoclasts formed by differentiation from CD14+ monocytes in the presence of 2-DG or glucose (0.01–10 mM). n = 8. *p < 0.05; **p < 0.01; ***p < 0.001.

Although there is considerable overlap in the genes regulated by HIF-1α and HIF-2α, there are also cell-type and microenvironmental differences^4^. Treatment of mature osteoclasts with isoform-specific *HIF* siRNA had no effect on basal levels of glucose uptake. *HIF-1α* siRNA prevented the hypoxic increase in glucose uptake related to increased glycolysis in hypoxic cells, while *HIF-2α* siRNA had no effect (Fig. 4a). 2-deoxy-d-glucose (2-DG) is an analogue of glucose that inhibits glycolysis following its phosphorylation to non-metabolizable 2-DG-6-P by hexokinase (HK)^34^. Treatment of mature osteoclasts with 2-DG dose-dependently prevented the hypoxic increase in osteoclast-mediated bone resorption without affecting osteoclast number (Fig. 4b,c), suggesting that inhibition of HIF-1α-induced glycolysis in mature osteoclasts could drive effects of *HIF-1α* siRNA to inhibit hypoxic bone resorption. In contrast to *HIF-1α* siRNA, 2-DG also inhibited osteoclast-mediated resorption of bone in normoxic conditions (Fig. 4d,e) and had a striking effect on osteoclast differentiation (Fig. 4f).

## Discussion

This is the first description of the induction of HIF-2α protein during the differentiation of human osteoclasts. *HIF-2α* siRNA had moderate effects on osteoclast differentiation but did not affect either osteoclast-mediated bone resorption or glucose uptake. This is the opposite phenotype to that observed with *HIF-1α* siRNA (no effect on differentiation, inhibition of hypoxic bone resorption and glycolysis), suggesting different roles for the two HIF isoforms in osteoclast biology.

Although there is a substantial body of work describing direct and indirect effects of hypoxia and HIF-1α on osteoclast formation and function, we could find only two references describing expression of HIF-2α in osteoclasts in vivo. Lee et al.observed immunohistochemical expression of HIF-2α in murine osteoclasts^8^. We described immunohistochemical expression of HIF‐1α and HIF‐2α in multinucleated osteoclasts within GCTB. Nuclear expression of HIF‐2α was observed in osteoclasts in 26/124 tumours, the presence of which was associated with expression of HIF-regulated BNIP3 and Glut‐1 in osteoclasts within the same tumour^27^.

Our current observation that HIF-1α and HIF-2α are present in the osteoclasts of some osteolytic tumours in the absence of marked expression of HIF in surrounding stromal cells led us to investigate whether HIF is induced during the differentiation of human osteoclasts. We have previously shown that *HIF-1α* and *HIF-2α* mRNA is induced during the differentiation of human CD14+ monocytes into osteoclasts using M-CSF and RANKL^17^. RANKL has variably been described to either upregulate *Hif-1α*, but not *Hif-2α*, mRNA via activation of NF‐κB^19,30^ or to increase the level of HIF-2α, but not HIF-1α, protein^8^ in murine osteoclasts. M‐CSF induces expression of HIF‐1α protein in human monocytes and osteoclasts and can induce detectable levels of HIF-1α and HIF-2α in MG-63 osteosarcoma cells^17,27^. As the level of detectable HIF-2α is very low in human osteoclasts, even under hypoxic conditions, it is possible that M-CSF also stabilises HIF-2α in these cells at a level below the detectable limit. We were able to detect low levels of HIF-2α protein in mature day 10 osteoclasts, as well as stabilisation of HIF-1α from differentiation day 4. Expression of HIF in osteoclasts in vivo could be affected by other osteoclastogenic growth factors^35^. For example, hepatocyte growth factor (HGF) can substitute for M-CSF to induce human osteoclast formation^36^. HGF can increase the translation of HIF-1α^37^ and induces HIF-1α protein in human osteoclasts^27^. Similarly to M-CSF, it is also able to stabilise HIF-2α in MG-63 cells^27^ and lack of effect in osteoclasts may be an issue with detection levels.

*HIF-2α* siRNA moderately reduced the number of osteoclasts formed during osteoclastogenesis in vitro. This is in agreement with Lee et al., who showed that over-expression of *Hif-2α* in murine BMMs enhanced osteoclast differentiation whereas inhibition of HIF-2α reduced osteoclastogenesis^8^. It is of interest that the effect of *HIF-2α* siRNA was different when osteoclastogenesis was performed in 3D, rather than 2D, culture; resulting in an increase, rather than a reduction, in the number of osteoclasts formed. It is not uncommon for cells cultured in 3D to exhibit altered sensitivity to cytokines, inhibitors or drugs^33,38,39^ or a stronger differentiation response^38,40^, but we can find no other reports of a reversal in the direction of effect of a stimulus due to 3D culture. It is possible that this relates to the microenvironment- and tissue-specific nature of the regulation and phenotypic effects of HIF-2α^4,5^ and is an area of considerable interest for future study.

Only *HIF-1α* siRNA prevented the hypoxic increase in osteoclast-mediated resorption of bone and neither *HIF-1α* nor *HIF-2α* knockdown affected normoxic levels of resorption. This is at first sight in contradiction to the report by Lee et al., who showed that over-expression of *Hif-2α* in murine BMM cells during differentiation caused increased F-actin ring formation and mineral resorption, whereas the opposite effect was seen in BMMs from *Hif-2α*^+*/−*^ mice^8^. However, we exposed only mature osteoclasts to *HIF* siRNA during this experiment, meaning that our data represents a direct effect of the HIF isoforms on resorption rather than potentially being a side-effect of phenotypic changes during osteoclast differentiation.

It is interesting to speculate whether the ability of digoxin to inhibit both osteoclast differentiation and hypoxic bone resorption by mature osteoclasts is due to its ability to inhibit both isoforms of HIF. Of note, the degree of inhibition of osteoclast differentiation by digoxin was much greater than that observed with *HIF-2α* siRNA. This could be due to non-HIF-mediated effects of this non-specific inhibitor. It might also be due to the ability of digoxin to inhibit HIF-regulated processes crucial to osteoclast formation and function to a level below the normoxic baseline.

Glucose metabolism is a key regulator of osteoclast-mediated bone resorption^41–43^ and osteoclasts promote interactions between glycolytic enzymes and components of the resorption machinery in order to micro-compartmentalise glycolytic ATP generation at intracellular sites where it can directly support resorption of bone^44–46^. We have described multiple mechanisms whereby HIF-1α stimulates glycolysis under hypoxia in order to drive increased bone resorption^47,48^ but, as shown in the current study, although *HIF-1α* siRNA inhibits hypoxic glucose uptake in osteoclasts, knockdown of neither *HIF* isoform affected glucose uptake in basal normoxic conditions.

The glucose analogue 2-DG inhibits glucose consumption and glycolysis in both basal and hypoxic conditions. It competitively inhibits glucose uptake because both sugars are transferred intracellularly by glucose transporters such as GLUT-1. Non-metabolizable 2DG-6-P then accumulates and inhibits both HK and glucose-6-phosphate isomerase (GPI) to prevent glycolysis^34^. 2-DG dramatically inhibited the hypoxic increase in bone resorption by osteoclasts, but also reduced basal levels of bone resorption and strongly inhibited osteoclast differentiation under normoxic conditions. Energy for osteoclast differentiation generally derives from mitochondrial oxidative metabolism, rather than glycolysis^46,49–51^. However, because 2-DG inhibits critical steps at the beginning of glucose metabolism both glycolysis and oxidative phosphorylation are disrupted, leading to decreased ATP production^34^.

Digoxin decreases mRNA levels of glycolytic enzymes including *Hk-1*, *Hk-2*, *Gpi* and *Pgk1*^31,52^, inhibits the activity of glucose-6-phosphate dehydrogenase, 6-phosphogluconate dehydrogenase and glutathione reductase^53^ and reduces glucose uptake^52^ in normoxic conditions, as well as in hypoxia. This could potentially reduce the efficacy of mitochondrial metabolism downstream of glycolysis, to explain its large effects on osteoclastogenesis as well as bone resorption. However, some reports indicate that digoxin actually improves mitochondrial metabolic function^54^ potentially implicating other digoxin-affected pathways in mediation of this anti-osteoclastogenic effect.

In summary, both HIF-1α and HIF-2α proteins are stabilised during the differentiation of human osteoclasts in vitro. The two HIF isoforms play distinct roles in osteoclast biology; HIF-2α modulating osteoclast differentiation and HIF-1α driving the hypoxic increase in bone resorption. Chemical inhibition of glycolysis can magnify the glycolysis-driven inhibitory effects of *HIF* knockdown on osteoclast formation and function, due to additional impact on basal glycolytic activity which causes reduced normoxic bone resorption and inhibition of osteoclast formation. In an in vivo setting, modulation of either isoform of HIF would also impact other cells within the bone microenvironment and their interactions with osteoclasts. We recently showed that the PHD enzyme inhibitor FG-4592 stabilises HIF-1α and HIF-2α, inhibits the differentiation of human monocytes into osteoclasts and stimulates osteoclast-mediated bone resorption. However, co-culture with osteoblasts amplified the inhibition of osteoclastogenesis and dampened the increase in bone resorption^33^. This study further opens the door to understanding the differential effects of HIF-1α and HIF-2α on osteoclast biology but acknowledges the complexities that will be involved in studying these differential effects in vivo.

## Methods

### Immunohistochemistry

Antigen retrieval of deparaffinised sections was performed by microwaving in EDTA (1 mM, pH 8). Sections were exposed to anti-HIF-1α (clone 54; BD Biosciences), anti-HIF-2α (EP190b, Abcam, Cambridge, UK), anti-Glut-1 (Abcam) or a serum control. Staining was visualized with the VECTASTAIN Elite ABC Kit with DAB (Vector Laboratories). Osteoclasts in tissue sections were considered as large, multinucleated cells containing ≥ 3 nuclei. Use of GCTB and osteosarcoma tissue sections from the Nuffield Orthopaedic Centre was approved by the Oxford Clinical Research Ethics Committee (C01.071). Samples and/or data obtained were collected with informed written donor consent in full compliance with national and institutional ethical requirements, the United Kingdom Human Tissue Act and the Declaration of Helsinki.

### Osteoclast culture

CD14+ monocytes were selected from the peripheral blood mononuclear cells of leucocyte cones (NHS Blood and Transplant, UK) by positive selection using magnetic microbeads. CD14+ monocytes were seeded in α-MEM (no ribonucleosides/deoxyribonucleosides, FBS (10%), L-glutamine (2 mM), penicillin (50 IU/ml), streptomycin sulphate (50 μg/ml)) onto either dentine discs or plastic dishes at 0.25 × 10^6^ (96 well plate) or 1 × 10^6^ (24 well plate) cells/well. For 3D culture, 1 × 10^6^ CD14+ monocytes were resuspended in 300 µl of collagen type I (2 mg/ml) in a 24 well plate and polymerisation was initiated by incubation at 37 °C for 30 min. Osteoclastogenesis was stimulated using M-CSF (25 ng/ml) and RANKL (30 ng/ml), with media and cytokines replenished every 3–4 days for 9 days. Cells were released from 3D culture using collagenase type I (0.2 mg/ml). Released osteoclasts were resuspended in α-MEM and re-seeded onto cell culture plastic for 16 h to allow adhesion prior to quantification. Hypoxia (2% O_2_, 5% CO_2_, balance N_2_) was initiated using a MiniGalaxy incubator. Use of leucocyte cones was approved by the London-Fulham Research Ethics Committee (11/H0711/7).

### Osteoclast formation and activity assays

Formalin-fixed osteoclasts were stained for tartrate-resistant acid phosphatase (TRAP) using naphthol AS-BI phosphate and Fast Violet B salt. Multi-nucleated cells containing three or more nuclei were considered to be osteoclasts. Immunostaining for the osteoclast-specific vitronectin receptor (VNR) used an anti-CD51/61 primary antibody (clone 23C6, 1:400; Bio-Rad, Oxford, UK). Resorption tracks formed by osteoclasts on dentine discs were revealed using 0.5% toluidine blue. The resorption tracks were high-lighted on images of the dentines using Adobe Photoshop and the total resorbed area per dentine disc was quantified using ImageJ.

### *HIF* siRNA

Mature osteoclasts were transfected with siRNA (50 nM) targeting *HIF-1α* (nucleotides 1521–1541 of *HIF-1α* [NM001530]; sense 5′-CUGAUGACCAGCAACUUGAdTdT-3′, antisense 5′-UCAAGUUGCUGGUCAUCAGdTdT-3′), *HIF-2α* (nucleotides 1260–1280 of *HIF-2α* [NM001430]; sense 5′-CAGCAUCUUUGAUAGCAGUdTdT-3′, antisense 5′-ACUGCUAUCAAAGAUGCUGdTdT-3′) or a scrambled siRNA control using RNAiMAX (Invitrogen). Duplexes were removed after 4 h and osteoclasts incubated for a further 16 h before exposure to experimental conditions. For osteoclast differentiation assays, cells were transfected with siRNA every 72 h.

### Western blotting

Osteoclasts were sonicated in lysis buffer (6.2 M urea, 10% glycerol, 5 mM dithiothreitol, 1% sodium dodecyl sulphate, protease inhibitors) before cell extract was separated by 8% SDS-PAGE and transferred onto a PVDF membrane. Membranes were probed with primary antibodies specific for HIF-1α (clone 54, 1:1000; BD Biosciences, Oxford, UK), GLUT1 (ab14683, 1:2500; Abcam, Cambridge, UK), LDHA (NBP1-48336, 1:2000; Novus Biologicals, Cambridge, UK) or β-tubulin (clone TUB2.1, 1:2500; Sigma-Aldrich, Dorset, UK). The chemiluminescent signal was detected using a UVITEC Alliance Q9 gel doc system and associated image acquisition software.

### Luciferase assay

A PGK HRE–firefly luciferase plasmid (obtained from Professor AL Harris, Oxford, UK) and a transfection control pHRG–TK renilla luciferase plasmid (Promega, Southampton, UK) were transfected into mature (day 8) osteoclasts using Lipofectamine 2000 (Invitrogen, Paisley, UK). Osteoclasts were exposed to hypoxia (2% O_2_) and/or 25–400 nM digoxin 24 h post-transfection. The Dual-Luciferase Reporter Assay System (Promega) was used to measure luminescence within cell lysates, normalising firefly luciferase luminescence to the renilla control.

### Glucose uptake

Glucose uptake was measured using the Glucose (GO) Assay Kit (Sigma Aldrich).

### Statistics

Results are derived from at least three independent experiments. Data are presented as mean ± standard deviation and were analysed using GraphPad Prism. Statistical analysis comprised one-way or two-way ANOVA using Dunnett’s or Tukey’s multiple comparison as a post-hoc test. Results were considered significant at p < 0.05.

## Supplementary information

Supplementary Information.

## Data Availability

All data generated or analysed during this study are included in this published article.
