# Supplementary material for: Distinct roles for the hypoxia-inducible transcription factors HIF-1α and HIF-2α in human osteoclast formation and function
Source: Sci Rep. 2020 Dec 3;10:21072. doi: 10.1038/s41598-020-78003-z (PMC7713367; doi:10.1038/s41598-020-78003-z)
Supplement: Supplementary file 1 — Supplementary Information. [file 41598_2020_78003_MOESM1_ESM.docx]

**Distinct roles for the hypoxia-inducible transcription factors HIF-1α and HIF-2α in human osteoclast formation and function**

Helen J Knowles

**
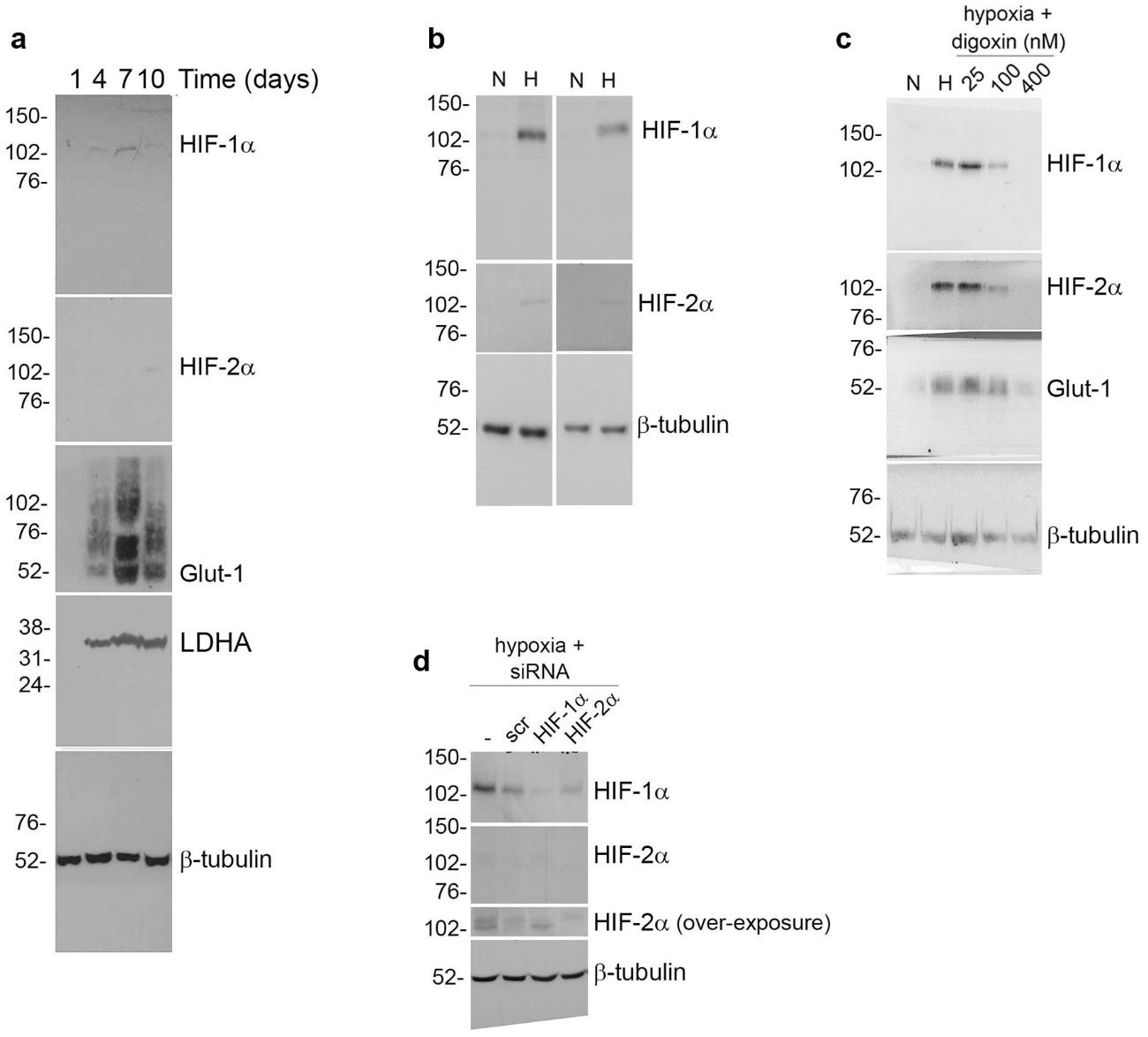
**

**Supplementary Figure 1.** Expanded Western blots relating to: (a) Figure 1b, (b) Figure 1c, (c) Figure 2a and (d) Figure 3a. Where full-length blots are not shown for HIF-1α or HIF-2α, the original blot was cut for detection of other low molecular weight proteins.
